# Housekeeping Queries

**Published:** 1912-07-06

**Authors:** 


### INSTITUTIONAL HOUSEKEEPING.

### Housekeeping Queries.

### How to Make Devonshire Cream.

"D. M."—*Our institute has a home farm attached to it, and I superintend the dairy. Devonshire clotted cream is required once a week, but, though I am well up in butter-making, I have never prepared the latter. Could you describe the process?*

It is very simple. Directly the milk comes in, strain it into open pans holding about 6 to 12 inches depth of milk. Stand these pans in a cool dairy for ten to twenty hours, according to the heat of the weather; then carry them steadily and put them over a moderate heat on the side of the stove. Heat gradually to about 175° F., when a crinkled ring appears and spreads over the surface. This heating should take not less than half an hour if the correct flavour is to be obtained. Next carry the pans back to the dairy and stand them on bars so as to allow for a current of air to pass under them that they may cool down as quickly as possible. Allow the pans to stand for some hours until perfectly cold, and then remove the thick, almost solid, cream with a perforated skimmer.

### Choice of Linen and Cotton Goods.

"£ s. d."—To distinguish between linen and cotton material examine the threads; the linen thread is stronger, flatter, and smoother, whereas the cotton breaks easily, is rounder, and has a fluffier surface; often the material has linen threads for the warp and cotton for the woof, so that the threads in both directions must be examined if linen material is being bought, and careful observation through a magnifying-glass is necessary, as cotton is often manufactured to look like linen. To ascertain if the material is firm and close it must be rubbed between the fingers, when, if good, there is little change in the fabric, and when held up to the light it must not look thin. If cotton material is bought, both sets of threads must be of good quality, or it is likely to split. Unbleached-cotton material is cheaper and wears better. This applies to bed linen, towels, tea-cloths, etc.; and it quickly becomes white after washing two or three times. Unbleached damask for table-cloths and serviettes can be bought by the yard of different widths and cut as required. Table-linen damask should be chosen of a good quality, firm and thick, for it will then last clean much longer, will not crush as easily, and gives the table a better appearance.

### Recipe for Poor Man's Cake.

Kitchen Matron.—*I should be grateful to be told how to prepare a very cheap cake—ours is a rescue home and very poor, but we wish to give cake once a week.*

This recipe is a good one used in several workhouses, and will, I think, answer your purpose. Slightly warm 4 oz. of dripping; beat into it 4 oz. of moist sugar until the mixture is very soft and creamy. Mix together 15 oz. of flour, one teaspoonful of salt, and ½ oz. of baking-powder. Add these to the dripping, etc. Add next 3 oz. of currants and about 2 gills of milk or milk and water. Beat well together and turn into a well-greased deep baking tin, and bake in a moderate oven for about half an hour. Product, 2 lb. Cut into slabs. Add spice if wished.

### How to Make Albumen Water.

Military Hospital.—The preparation of the albumen water as you describe it is incorrect. Try this method. Put the white of an egg into a basin, beat it to a light froth. Add five to seven ounces of water, and, if allowed, a little salt or a few drops of strained lemon-juice for flavouring. Beat together, then cover and leave until the froth has subsided, when it is ready to administer to the patient. Sometimes stimulants are also added. You are correct in thinking that this preparation is frequently ordered in cases of diarrhœa, etc., in place of raw beef-juice.

### Twice-Cooked Meat.

Puzzled.—*Will you kindly tell me why twice-cooked meat is injurious, or less nourishing, and what happens to it to detract from its value during the second cooking? I am obliged to utilise cold meat, and it is not popular with my staff unless it is cooked up again and served hot.*

Twice-cooked meat is not injurious unless it is heated in such a fashion that it is rendered hard and leathery, in which case undue strain is thrown on the digestive organs. This is specially unfortunate when it occurs with individuals who are physically or mentally fatigued. Cold meat re-heated is less nourishing, because, no matter how careful the cooking process, a certain amount, however small, of the nutritive juices escapes during the first cooking, though retained and served as gravy. When the meat is re-heated, this gravy being used and the juices which have escaped from the cut surfaces into the dish having dried, the meat contains not only less flavour, but also less nourishment. The cook to recompense these losses adds, if she is a wise woman, various piquant flavourings, extra sauces and so forth, producing finally a very savoury and easily digested concoction, but still one less actually nourishing than a freshly-cooked steak or cut from a joint supposing they had been correctly prepared in the widest sense of the term. In cases where meat has in the first instance been stewed to rags, or badly over-roasted, it has but a low food value, even when first served. It is necessary to bear in mind that meat should never be twice *cooked;* it requires not cooking the second time, but merely re-heating. It is the faulty method of utilising cold meat that has really won for it such a bad name. Meat when re-heated, no matter whether it be in a mince, hash, curry, pie, or rissole, must never quite reach simmering point, certainly not 212° F. Bear these points also in mind: Use stock made from the bones and rough bits for moistening purposes and never water; supplement a made-up dinner with a specially-nourishing pudding and well-cooked vegetables, and you need not fear that you are doing your staff an injustice.

### The Choice of Meat.

Assistant Housekeeper.—*We are not satisfied that our contractor is treating us fairly. Can you give me any signs by which to distinguish between English and foreign meat, or to recognise whether the meat is really satisfactory in quality when it is sent in every day?*

We fear that any information we might attempt to give on this subject would be unintelligible to one without the technical knowledge which can only be learned from an expert. The appearance of meat varies so much according to variations in temperature, and in the case of foreign meat is so much altered in the process of thawing that it is practically impossible to give data upon which reliable conclusions could be arrived at. If your institution is a large one it would be worth while to adopt a system much in vogue in the North, and give a retaining fee to a reputable retired butcher whose business it would be to inspect the goods delivered each morning, rejecting anything not according to contract, and reporting weekly or monthly to the board of management. In many localities it would be possible to get honorary service of this kind.

### NOTICE TO MATRONS.

We are always pleased to answer queries and give recipes which may be asked for by our readers. Let us know if any difficulties are hampering you, and we will do our best to find a remedy. Any successful experiments you may be making in culinary matters would interest a wide circle. We should be specially glad to hear particulars of any scheme for nurses' diet which works out at 6s. a week.

